# Modified Center-Edge Angle in Children with Developmental Dysplasia of the Hip

**DOI:** 10.3390/jimaging11010003

**Published:** 2024-12-27

**Authors:** Katharina S. Gather, Fabian Sporer, Christos Tsagkaris, Marco Götze, Simone Gantz, Sebastien Hagmann, Thomas Dreher

**Affiliations:** 1 Clinic for Orthopedics and Trauma Surgery, Center for Orthopedics, Trauma Surgery and Spinal Cord Injury, Heidelberg University Hospital, Schlierbacher Landstrasse 200a, 69118 Heidelberg, Germany; fsporer92@gmail.com (F.S.); marco.goetze@luks.ch (M.G.); simone.gantz@web.de (S.G.); sebastien.hagmann@atos.de (S.H.); 2 Departement für Pediatric Orthopedics, University Hospital Mannheim, Theodor-Kutzer-Ufer 1-3, 68167 Mannheim, Germany; 3 Clinic Böblingen, Clinic for Pediatric Medicine, Bunsenstarße 120, 71031 Böblingen, Germany; 4 Department Pediatric Orthopaedics and Traumatology, Children’s University Hospital, Lenggstrasse 30, 8008 Zürich, Switzerland; christos.tsagkaris@kispi.uzh.ch (C.T.); thomas.dreher@kispi.uzh.ch (T.D.); 5 German Joint Center Heidelberg, ATOS Clinic, Bismarckstrasse 9-15, 69115 Heidelberg, Germany; 6 Balgrist Orthopaedic University Hospital, Pediatric Orthopedics, Forchstrasse 340, 8008 Zurich, Switzerland

**Keywords:** CE angle of Wiberg, modified CE angle, developmental hip dysplasia

## Abstract

Developmental dysplasia of the hip (DDH) is a prevalent developmental condition that necessitates early detection and treatment. Follow–up, as well as therapeutic decision-making in children younger than four years, is challenging because the center–edge (CE) angle of Wiberg is not reliable in this age group. The authors propose a modification of the CE angle (MCE) to achieve comparable reliability with the CE among children younger than four and set diagnostic thresholds for the diagnosis of DDH. 952 anteroposterior pelvic radiographs were retrospectively reviewed. The MCE is defined on X-ray pelvic overview images as the angle between the line connecting the epiphyseal joint center and the outer edge of the acetabulum, and perpendicular to the Hilgenreiner line. The MCE angle exhibited high sensitivity and specificity, as well as intrarater variability comparable to the CE among children younger and older than four years. The authors recommend cut-off values for the MCE angle; for children under four years old, the angle should be equal to or greater than 15 degrees; for those under eight years old, it should be equal to or greater than 20 degrees; and for those eight years old and older, it should be equal to or greater than 25 degrees. However, the MCE angle’s reliability diminishes around the age of nine due to the curvature of the growth plate, which complicates accurate measurement. This study showed that the MCE angle can be used adequately in children under four years and could be used as a progression parameter to diagnose DDH.

## 1. Introduction

Developmental dysplasia of the hip (DDH) is a developmental condition where the hip joint does not form properly, resulting in the femoral head not being adequately covered by the acetabulum. This anatomical configuration leads to hip and groin pain, gait alterations, femoacetabular impingement, and precipitates hip osteoarthritis as a result of continuous low-grade injury to the joint components [1]. A number of anteroposterior radiographical measurements have been devised to quantify the acetabular coverage of the femoral head and subsequently guide surgical decision-making and postoperative evaluation. These include, but are not limited to, the Migration Index (MI), the Acetabular Index (AI) or Tönnis angle, the acetabular (AC) angle, and the center–edge (CE) angle [2,3].

The CE angle, first described in 1939 by Gunner Wiberg, assesses the lateral coverage of the femoral head, quantifying henceforth the degree of acetabular deficiency in DDH. Formed between a vertical line drawn through the center of the femoral head and a line connecting the center of the femoral head to the lateral acetabular edge, the CE angle provides a measurement of the depth and development of the acetabulum relative to the femoral head position among children and adults. The physiological values of the CE angle exhibit an age-dependent variation, but in principle, angles exceeding 20 and 25 degrees are respectively acceptable among children between 5 and 8 years and individuals older than 9 years [4,5,6,7].

Although a wealth of studies consider the CE angle to be a reliable and reproducible measure, its application exhibits a number of practical limitations. Defining the center of the femoral head when its appearance is delayed or eccentrically located is challenging and might lead to misleading measurements [8,9]. This is particularly relevant in patients with congenital dysplasia, avascular necrosis of the femoral head (Legg-Calve-Perthes disease), slipped capital femoral epiphysis (SCFE), and other conditions resulting in a deformity of the femoral head [9]. Similar limitations concern the Tönnis classification, which also depends on the existence and position of the center of the femoral head [8].

The applicability of the CE angle faces additional age-related limitations. Originally validated for patients between 8 and 75 years and later deemed reliable for the age group of 5–8, the CE angle cannot be used for younger patients due to the inadequate formation of the femoral head among them [5,10]. The Acetabular Index (AI), an angle formed between a line drawn along the weight-bearing surface of the acetabulum and a horizontal line parallel to the inter-teardrop line, is considered reliable in this age group. Transitioning between two different indexes across child growth can pose challenges to the consistency of follow-up and the integrity of therapeutic decision-making.

Expanding the application of the CE angle to younger children could resolve this potential discrepancy, enabling clinicians to fully utilize the growing number of conservative and surgical treatment options. To achieve this goal, it would be necessary to replace the center of the femoral head with an anatomical landmark that can be reliably identified in children younger than 4 years, such as the midpoint of the proximal epiphyseal growth plate of the femur. The latter is already used in the hip dysplasia classification of the International Hip Dysplasia Institute (IHDI), rendering it applicable to all ages [8].

The authors have previously proposed the modification of the CE angle by replacing the center of the femoral head with the midpoint of the proximal epiphyseal growth plate of the femur. The modified CE angle has been shown to have comparable intra- and interobserver reliability with the CE angle of Wiberg [11]. On this basis, the authors aim to (1) evaluate the differences between the original and the modified CE angle and to define the physiological thresholds of the latter in a large patient cohort, and (2) investigate the reliability and validity of the modified CE angle for children younger than 4 years.

## 2. Materials and Methods

### 2.1. Patients

Patients were recruited retrospectively through the digital medical records of the Orthopedic Surgery Department of a tertiary university hospital after the study protocol had been reviewed by the Ethics Committee of the Medical Faculty affiliated with the same university hospital. Patients aged 0–18 years who had undergone a pelvic anteroposterior radiograph between 2010 and 2018 and were diagnosed with congenital hip dysplasia or had an unremarkable X-ray were considered eligible for inclusion in the study. A total of 1820 patients with 5339 pelvic radiographs, i.e., 10,678 hip joints, were evaluated on this basis. Candidates for inclusion were excluded in case (a) the quality of the images was inadequate for the measurement of the CE angle, the plane or the rotation of the image was different from the anteroposterior projection of the pelvis, patient records were incomplete (n = 592), (b) the growth plate was not recognizable (n = 3108), (c) patients had undergone hip surgery prior to the initial presentation (n = 106), (d) patients presented with other diseases and syndromic conditions associated with hip deformity such as Perthes’ disease or femoral head necrosis (n = 162), as well as (g) neurogenic hip dysplasia (n = 838). To prevent bias from patients with multiple follow-up images, only one image per patient per age group was evaluated, resulting in additional exclusions (n = 1398).

Ultimately, 952 radiographs and 1904 hip joints were included in the evaluation of the modified CE angle.

The following data were collected for each patient: age, gender, side, and complications, if any.

### 2.2. Methods

Radiographs of children without any previous surgery were analyzed. Measurements were made on anterior-posterior pelvic X-rays, during the acquisition of which patients were lying on their backs with a slight internal rotation of the leg so that the big toes touched one another. All radiographs were analyzed by a student, and 100 radiographs were also analyzed by a resident and an orthopedic consultant to evaluate inter- and intraobserver reliability.

The CE angle according to Wiberg, the modified CE angle, the Acetabular Index (AI), the projected collum-diaphyseal angle (CCD angle), the Tönnis degree, and the Migration Index were designed on each radiograph.

The CE-angle of Wiberg was measured as formerly described [5,10]. Briefly, the angle was defined as the angle between a line from the center of the femoral head to the lateral bony edge of the acetabulum and a perpendicular line through the center of the femoral head. The center of the femoral head was first obtained using a circle that outlined the femoral head. The perpendicular line was adjusted to be rectangular to Hilgenreiner’s line.

**Figure 1:**
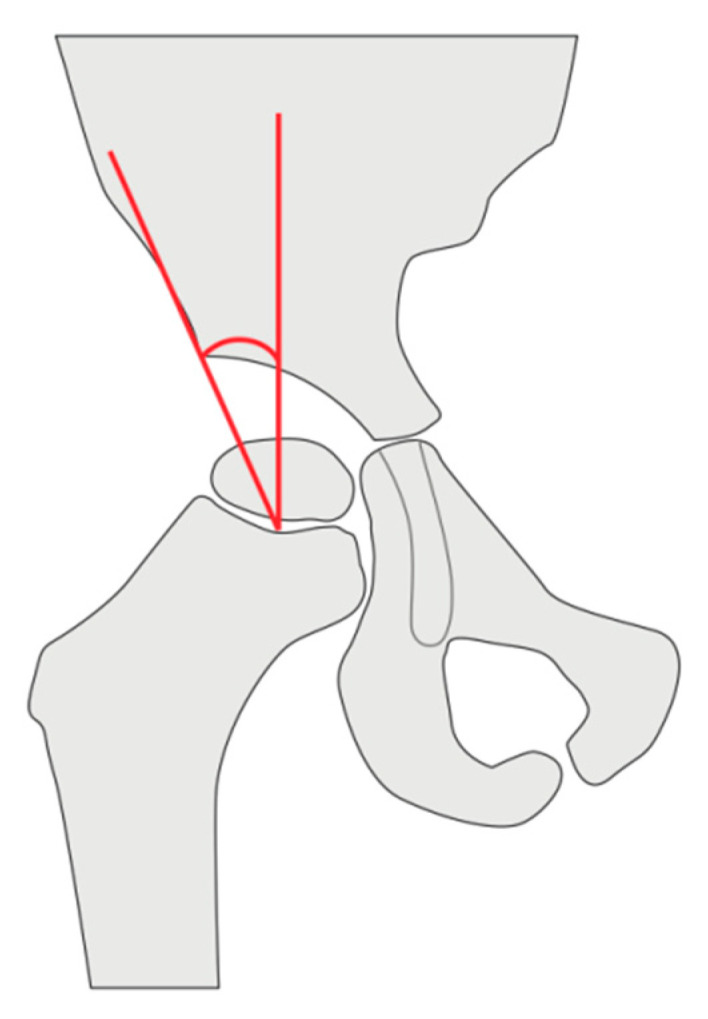
Measurement of the modified CE-angle. Instead of the center of the femoral head, the middle of the growth plate was used as a reference. The modified CE-angle is then measured between the perpendicular line that is rectangular to Hilgenreiner’s line (right red line) and the line crossing the lateral bony edge of the acetabulum (left red line).

The modified CE-angle was measured in the same way, except that instead of referring to the center of the femoral head, the center of the growth plate (measured and halved) was used as a reference (Figure 1).

### 2.3. Statistical Analysis

The statistical analysis was conducted with SPSS Statistics (version 22.0; SPSS, Chicago, IL, USA) and Microsoft Excel separately for physiological and pathological hips. *p* values < 0.05 are treated as statistically significant. The correlation coefficient according to Bravais and Pearson has been used to determine whether there is a relationship between two values. The von Bland-Altmann test was used to compare the agreement between several measurement methods and also to include the scattering and distortion of the measured values.

The correlation analyses, along with Cronbach’s alpha, were employed to assess the inter- and intraobserver reliability of the measurements. Additionally, these analyses were utilized to compare the modified CE angle with the traditional CE angle and the AI, ensuring the comparability of these metrics within the study. The receiver operating characteristic (ROC) curves were used to determine new cut-off values for the modified CE angle. The area under the curve (AUC) indicates the discriminatory power of the test, and the location of the greatest discriminatory power of the modified CE angle to distinguish between pathological values and healthy values is determined by calculating the Youden Index.

## 3. Results

A total of 952 pelvic overview images and 1904 hip joints were subjected to analysis. The analysis of both joints depicted in every single image was subject to symmetrical epiphyseal closure and X-ray protection placement; should one of these conditions not be satisfied, the joint whose analysis was compromised would be further excluded from the analysis. The evaluation was carried out in five age groups: 0–2 years, 3–4 years, 5–7 years, 8–9 years, and 10 years and older. The radiological measurements for each hip joint were classified as physiological or dysplastic based on the diagnostic information in the patient’s record, the AI or the measured CE. As a result, 957 hip joints were categorized as physiological and 947 as congenitally dysplastic.

### 3.1. Physiological and Pathological Values of the Modified CE Angle

**Figure 2:**
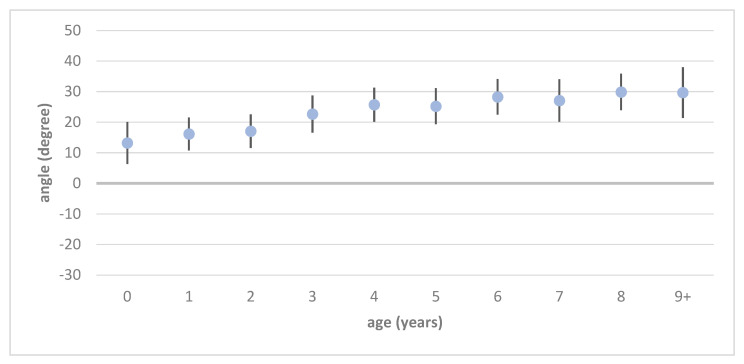
Mean value and standard deviation of the modified CE angle throughout physiological hip joints.

**Figure 3:**
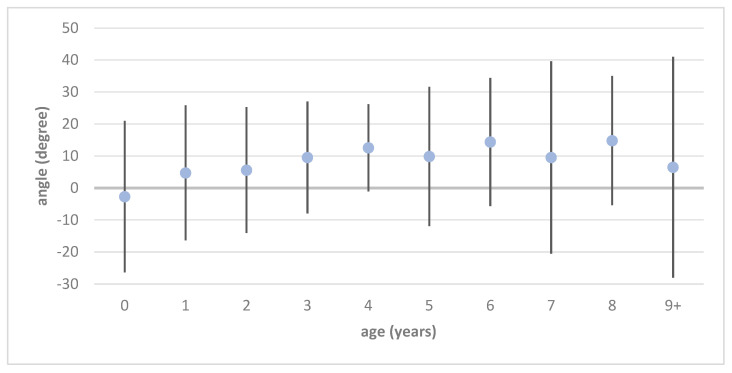
Mean and standard deviation of modified CE angles in the course of developmental dysplastic hip joints.

The values measured with the modified CE angle are visually presented in Figure 2 and Figure 3 and reported in detail in Table 1 and Table 2. Of the physiological hip joints, 527 were male and 430 were female. Of the developmental dysplastic hip joints, 296 were male and 651 were female. Unilateral DDH was found in 223 patients (110/223 right and 113/223 left), while the remaining 362 patients had bilateral DDH.

The values for the modified CE angle in children with physiological hip development are approximately 15° (16.14°, S = 5.39) in infancy and increase to approximately 30° (29.67°, S = 8.33) by the age of 9 years (see Figure 2 and Table 1). In developmental dysplastic hip joints, the modified CE angle increases to nearly 26° (25.21–25.72°, S = 5.9) in physiologically developed hips by 4–5 years of age, while in developmental dysplastic hips, stagnates at a range between 10 and 15° (see Figure 3 and Table 2).

### 3.2. Inter- and Intraobserver Reliability

Integrating the modified CE angle in clinical practice requires robust measurement results that do not exhibit notable variations across different measurements by the same or different operators, particularly clinicians at different levels of seniority and experience involved in the interpretation of the radiographs and relevant decision-making. The quality criteria for this are intraobserver reliability. In this regard, a very high correlation of at least >0.95 was consistently shown for both the CE angle according to Wiberg and the modified CE angle.

High interobserver reliability is also of great importance for the use of the modified CE angle in clinical practice. An intraclass correlation coefficient of 0.965 and a Cronbach’s alpha of 0.975 were found for the modified CE angle. A high correlation, with an intraclass correlation coefficient of 0.918 and a Cronbach’s alpha of 0.930, was also found for the CE angle according to Wiberg. This is also calculated explicitly for children aged 0–10. Overall, the interrater correlation in this case is slightly lower on average than with the modified CE angle, but not significant.

### 3.3. Defining Cut-Off Values for the Modified CE Angle

**Figure A1:**
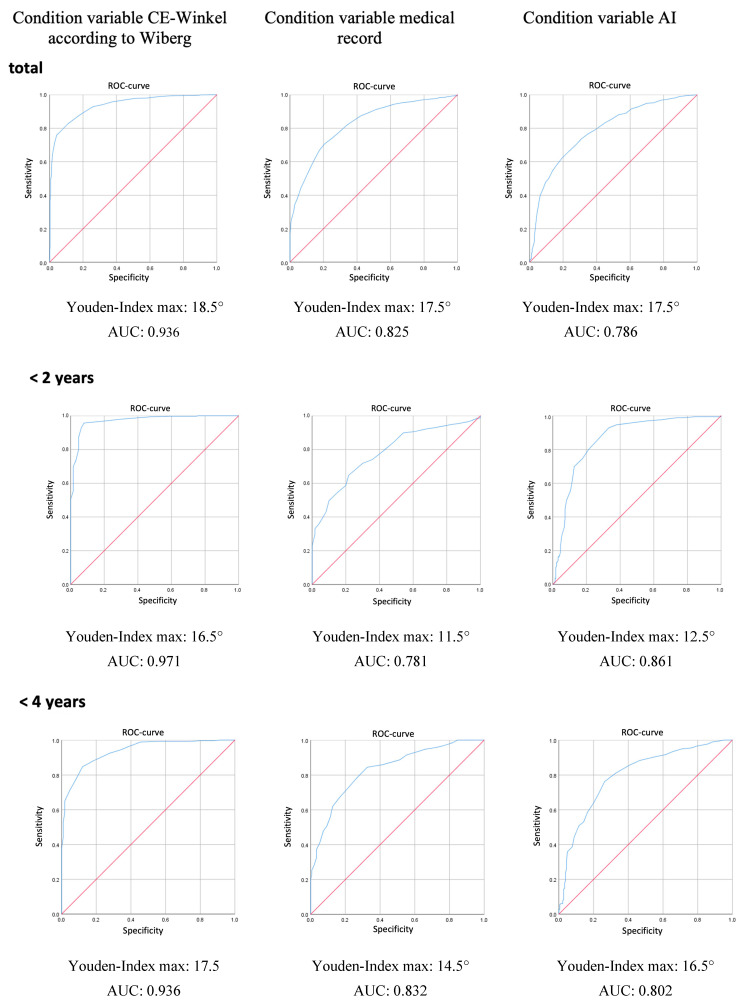

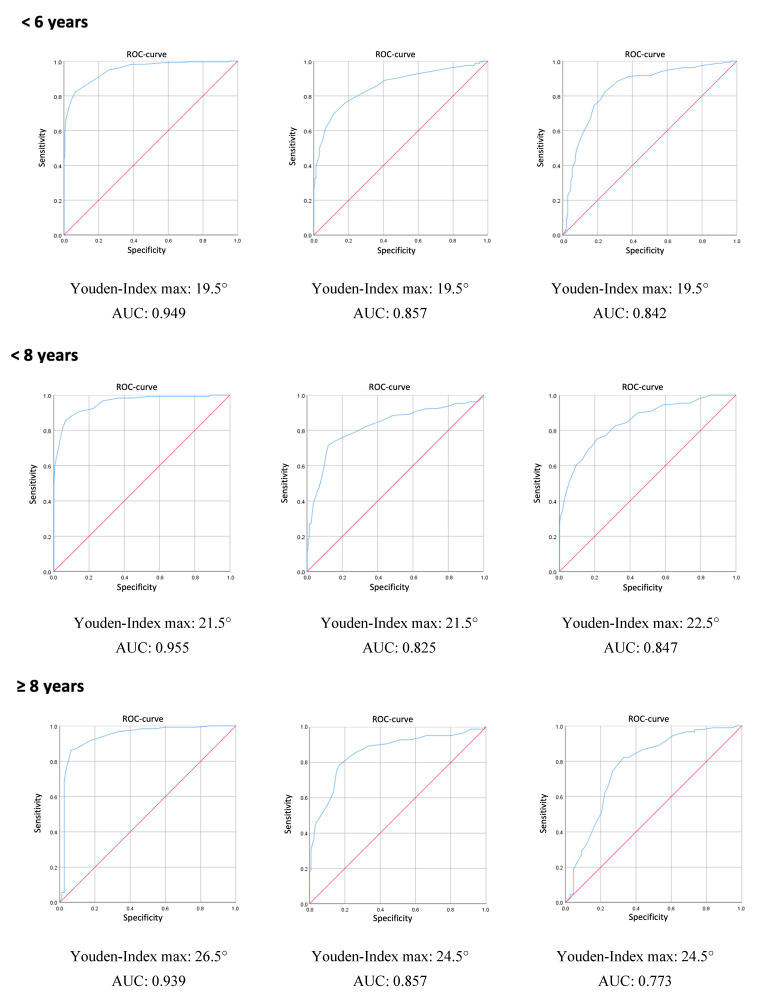
ROC curves, divided according to the corresponding condition variables CE angle according to Wiberg, medical record and AI as well as according to different age groups, each with the corresponding AUC and the respective Youden Index for developmental dysplastic hip joints.

Cut-off values appropriate for the distinction between physiological and dysplastic hip development with the modified CE angle are listed in Table 3, while their statistical calculation is visualized in Figure A1 (Appendix A).

All values for the modified CE angle were analyzed in the overall cohort and separated by gender. There were no significant differences between the genders; hence, common values for males and females are presented below.

As described, an ROC curve analysis was used. The decision as to whether the hip joint was healthy or dysplastic was made in three different ways: according to the CE angle, according to Wiberg, according to the medical records, or according to the AC angle. Figure A1 (Appendix A) shows the results for DDH separated by age group and by the condition variables CE angle according to Wiberg, medical records, and AC angle. This shows that the value for the ‘Area under Curve’ is above 0.7 for each state variable, which indicates a high quality of the modified CE angle as a test parameter.

Table 3 shows the cut-off values with the highest discriminatory power of the modified CE angle determined according to the Youden Index for developmental dysplastic hip joints.

Further integrating these values by age group, a cut-off value of 15.08° is obtained for children under four years of age, 20.67° for children under eight years of age, and 25.33° for children over eight years of age.

The following cut-off values for the modified CE angle are therefore recommended in this study for the children younger than 4 years, children between 4 and 8 years of age, as well as individuals older than 8 years:

<4 years:>15°
<8 years:>20°
≥8 years:>25°

To find out whether these standard values measure what they are supposed to measure, the sensitivity and specificity of the modified CE angle were measured using these standard values and compared with recognized angles, such as the classic CE angle according to Wiberg or the Acetabular Index (AI).

In Table 4, the test quality criteria are calculated for the CE angle according to Wiberg, the modified CE angle and the AC angle. The medical record, the CE angle, and the AC angle are used to determine the difference between healthy and pathological as condition variables. A positive test result means that the measurement method under investigation indicates a pathological value. The respective number of analyzed values for the total cohort and separately for those younger and older than 4 years can be found in Table 4.

In order to determine the test quality criteria for the modified CE angle, the AI should be used as the condition variable for those under four years, and the CE angle should be used as the condition variable for those four years and older. This decision was made to objectify the evaluation, given that the retrospectively recorded medical records had a higher chance of being affected by human error. For the overall cohort, this results in a sensitivity of 0.787, a specificity of 0.816, a positive predictive value of 0.809, and a negative predictive value of 0.794 for the modified CE angle.

## 4. Discussion

Advances in the diagnosis and surgical treatment of developmental hip dysplasia have enabled clinicians to perform open reduction or corrective pelvic and femur osteotomies in children younger than 4 years at the time of surgery. Evidence suggests that the average age at the time of surgery ranges from 1.8 to 4.5 years as of 2012 [12,13,14,15].

Radiographs are widely accepted as the basis of therapeutic decisions among children of walking age. To date, treatment decisions for children under 4 years of age have been made almost exclusively on the basis of the AI. Nevertheless, their interpretation in children younger than 4 years is challenging. Commonly used measurements such as the AI do not directly quantify the femoral head’s coverage. A growing body of evidence has questioned the reliability of the AI in children under the age of 3, which was previously thought to be high [16,17,18]. This stresses the importance of evaluation tools that enable surgical decision-making within this demographic.

The present study addresses the need for reliable methods to evaluate femoral head coverage [17,18]. In particular, it proposes a modified CE angle that can be effectively used for diagnostic evaluation and surgical decision-making in children younger than 4 years. Our proposed modification consists of replacing the femoral head—which is yet to be completely formed in this age group—with the epiphyseal joint center. This approach is consistent with the findings of Firth and colleagues (2016), who showed the prognostic potential of the ossific nucleus center edge angle (ONCEA) in patients below 5 years [17]. Furthermore, the potential of the ossification center of the hip as a landmark for the assessment of DDH and its sequelae has been recently discussed in studies focusing on avascular necrosis (AVN) [19,20]. Hence, the present study builds on the growing scientific discourse surrounding the epiphyseal joint center to propose a new method for measuring femoral head coverage in an age group where reliable measurements are essential.

### 4.1. Diagnostic Reliability of the Modified CE Angle

In children aged four years and younger, the sensitivity of the modified CE angle is 80.2%. This is comparable to the sensitivity of the CE angle of Wiberg at 81.3% for detecting hip dysplasia among older children, given that, to our knowledge, the classic CE angle has not so far been reportedly used in this age group. Further, this is comparable to the sensitivity of the classic CE angle at 84.2% for CAM hip deformity [21]. This study yielded a specificity of 65.9% for the modified CE angle, which is superior to the respective value of 37.7% for the CE angle of Wiberg. The positive predictive value is 66.4%, which is slightly lower but comparable to the classic CE angle (73.3%) and the negative predictive value in these measurements is 79.8%.

The modified CE angle is also suitable for the diagnosis and monitoring of hip dysplasia among children older than four, with a sensitivity of 77.8% and a specificity of 94.9%. Consequently, the modified CE angle can be considered adequate for the diagnosis of DDH in children younger and older than four.

It should be noted that the modified CE angle cannot outperform the reliability of the CE angle of Wiberg, since the latter serves as a state variable. Nevertheless, it performed comparably to proposed measurements based on the shape of the outer edge of the acetabular roof, the position of the femoral head, the Shenton-Menard arch, and the AI, with a sensitivity of 61% and a specificity of 87% [22].

Interrater reliability:

This study showed consistently high correlations between the results of clinicians with varying experience, with an intraclass correlation (ICC) of 0.965 for the modified CE angle, similarly to the classic CE angle reaching an ICC of 0.918.
Although the CE angle of Wiberg has shown good interrater reliability with an ICC of 0.88 [7] and interrater differences not exceeding 4.0° [23], studies where its ICC does not exceed 0.55, albeit being higher than other radiological parameters [24], support the satisfactory interrater reliability achieved by the modified CE angle.

### 4.2. Diagnostic Thresholds of the Modified CE Angle

To render the proposed modification of the CE angle useful in clinical practice, the authors averaged the findings in three major age groups and recommend a diagnostic cut-off of 15° for children younger than 8 years, 20° for children between four and eight years, and 25° for children of 8 years or older. However, it should be noted that the modified CE angle’s reliability diminishes around the age of nine due to the curvature of the growth plate, which complicates accurate measurement. Re-evaluating these thresholds in studies with larger populations, provided that they then continue to demonstrate high reliability and validity, can make their use in routine clinical practice feasible.

### 4.3. Limitations and Future Research

The study is subject to a number of limitations concerning its methodology and execution or the interpretation of its results. The latter primarily concerns the anatomical underpinnings of the measurements that are beyond the control of the researchers. Regarding the methodology, although the interrater validity was high, it should be noted that it was not possible to have the entire population measured thrice on organizational grounds; therefore, the performance of the operators was tested in randomly selected samples of the selected images representing all the age groups. Moreover, the measurement method was clearly specified, and the technique was meticulously taught to all the involved operators. This might not simulate the level of control over each other’s measurements outside of the study settings.

Interpretation-wise, the age-dependent specificity of the modified CE angle should be considered. The study shows considerably higher specificity in children older than four than those younger than four, but with approximately the same high sensitivity. Recognizing a physiologically developed hip in children younger than four is more challenging because of the premature anatomical development. This could lead to a different ruling of physiological and dysplastic hips with the Acetabular Index and the modified CE angle. The first measures the inclination of the acetabulum, while the latter measures the position of the (growing) femoral head in relation to the acetabulum. The measurements heavily rely on the exhibited ossification of the cartilaginous structures of the joint. Therefore, it is possible that the femoral head may not be optimally positioned with a physiological acetabulum, which would lead to a pathological modified CE angle.

Future research is essential to consolidate these results and mitigate the limitations of this study. Age-specific adaptations of the modified CE angle need to be explored to improve its accuracy across different age groups, particularly in children under four and those over nine, with a special focus on defining widely acceptable cut-off values for clinical practice. Scaling this measurement across patients with syndromic conditions and neurogenic hip dysplasia is also necessary as a following step. Within and across both groups, integrating these measurements in pre- and postoperative evaluation could provide further insights in the reliability of the proposed modification of the CE angle in surgical settings.

## 5. Conclusions

Overall, this study was able to show that the modified CE angle can be used adequately in children under the age of four and could be used as a follow-up parameter for diagnosing DDH. In addition, it is also comparable with the CE angle of Wiberg in the age group of 5–8 years.

However, due to the epiphyseal groove, the modified CE angle can only be used consistently and reliably up to the age of eight years.

## Figures and Tables

**Table 1:** Mean values and standard deviation of modified CE angles separated by age and gender for physiological hip joints.

Age	Male	ΔS	Female	ΔS	Total	ΔS
0	13.25	±7.29	13.13	±6.64	13.2	±6.86
1	16.41	±5.98	15.8	±4.58	16.14	±5.39
2	17.24	±5.61	16.88	±5.45	17.07	±5.49
3	23.98	±6.19	19.14	±4.26	22.65	±6.1
4	25.77	±6.43	25.67	±4.71	25.72	±5.57
5	24.98	±4.94	25.51	±7.02	25.21	±5.9
6	27.88	±4.98	28.57	±6.44	28.27	±5.83
7	27.27	±6.49	26.53	±8.09	27.06	±6.96
8	29.91	±6.00	29.8	±5.97	29.87	±5.95
9+	29.86	±7.55	28.71	±12.24	29.67	±8.33
total	24.23	±7.82	23.34	±8.14	23.88	±7.96

**Table 2:** Mean values and standard deviation of modified CE angles separated by age and gender for hip joints with developmental dysplasia of the hip.

Age	Male	ΔS	Female	ΔS	Total	ΔS
0	5.44	±15.68	−4.97	±25.22	−2.68	±23.69
1	2.42	±24.72	5.35	±20.02	4.73	±21.07
2	9.97	±6.72	3.93	±22.57	5.60	±19.67
3	8.81	±18.06	10.04	±17.21	9.54	±17.46
4	9.74	±16.35	13.62	±12.39	12.56	±13.63
5	3.13	±28.41	12.41	±18.17	9.85	±21.74
6	12.65	±23.91	15.67	±16.61	14.37	±19.99
7	12.32	±27.07	7.45	±32.49	9.55	±30.08
8	9.28	±26.30	20.32	±8.83	14.80	±20.20
9+	−1.42	±38.20	16.53	±27.12	6.50	±34.50
total	7.43	±23.26	8.84	±20.29	8.40	±21.26

**Table 3:** Values of the modified CE angle of greatest discriminatory power by age and condition variable, separately for developmental dysplasia of the hip.

Cut-Off-Values Modified CE Angle			
Classified by	CE	Record	AI
<2 age	17.5	11.5	12.5
<4 age	17.5	17.5	15.5
<6 age	19.5	19.5	19.5
<8 age	21.5	21.5	22.5
≥8 age	26.5	23.5	26.5
Total	18.5	17.5	14.5

**Table 4:** Values of the modified CE angle of greatest discriminatory power by age and condition variable separately for developmental dysplasia of the hip. Sens = sensitivity, Spec = specificity, PPV = positive predictive value, NPV = negative predictive value.

		CEM to Record	CE to Record	AI to Record	CEM to CE	AI to CE	CEM to AI	CE to AI
total	Sens	0.596	0.734	0.550	0.751	0.625	0.749	0.874
	Spec	0.736	0.597	0.764	0.955	0.850	0.727	0.577
	PPV	0.818	0.784	0.824	0.965	0.874	0.688	0.625
	NPV	0.477	0.530	0.458	0.699	0.577	0.783	0.850
<4 years	Sens	0.632	0.813	0.551	0.725	0.580	0.802	0.953
	Spec	0.618	0.377	0.745	0.972	0.913	0.659	0.418
	PPV	0.777	0.733	0.821	0.988	0.953	0.664	0.580
	NPV	0.443	0.490	0.439	0.538	0.418	0.798	0.913
≥4 years	Sens	0.568	0.674	0.550	0.778	0.672	0.709	0.813
	Spec	0.817	0.749	0.778	0.949	0.825	0.775	0.691
	PPV	0.856	0.837	0.827	0.945	0.813	0.711	0.672
	NPV	0.497	0.545	0.471	0.792	0.691	0.773	0.825

## Data Availability

The data supporting the outcome of this research work have been reported in this manuscript.
